# Patient Recruitment Strategies for Behavioral Clinical Trials in Adults with Inflammatory Bowel Diseases: An Analysis of the ADEPT (Addressing Disability Effectively with Psychosocial Telehealth) Randomized Controlled Trial

**DOI:** 10.1093/crocol/otaf033

**Published:** 2025-05-02

**Authors:** Kanika Malani, Chung Sang Tse, Sumona Saha, Megan Lutz, Sasha Taleban, Samir A Shah, Hannah Fiske, Melissa Hunt, Lily A Brown, Robert Kuehnel, Brittaney Bonhomme, S Alandra Weaver, Raymond K Cross, James D Lewis, Sara Nicole Horst

**Affiliations:** Brown University, Providence, Rhode Island, USA; University of Pennsylvania, Philadelphia, Pennsylvania, USA; University of Wisconsin System, Madison, Wisconsin, USA; University of Wisconsin System, Madison, Wisconsin, USA; Banner - University Medical Center South, Tucson, Arizona, USA; Brown University, Providence, Rhode Island, USA; Gastroenterology Associates, Inc., powered by GI Alliance, Providence, Rhode Island, USA; Brown University, Providence, Rhode Island, USA; University of Pennsylvania, Philadelphia, Pennsylvania, USA; University of Pennsylvania, Philadelphia, Pennsylvania, USA; University of Pennsylvania, Philadelphia, Pennsylvania, USA; University of Pennsylvania, Philadelphia, Pennsylvania, USA; Crohn’s & Colitis Foundation, New York, New York, USA; Melissa L. Posner Institute for Digestive Health & Liver Disease at Mercy Medical Center, Baltimore, Maryland, USA; University of Pennsylvania, Philadelphia, Pennsylvania, USA; Vanderbilt University Medical Center, Nashville, Tennessee, USA

## Abstract

**Background:**

This study evaluates the effectiveness of different methods to recruit patients with inflammatory bowel disease (IBD) into a randomized controlled trial (RCT).

**Methods:**

630 participants were recruited into a multicenter RCT using electronic medical record (EMR) bulk messaging, in-person study discussion with a clinician, or a hybrid method combining the above approaches.

**Results:**

Bulk EMR messaging alone had the highest recruitment and response rates, required the least amount of time to implement, and incurred the lowest cost as compared to the in-person and hybrid recruitment methods.

**Conclusions:**

Digital health technology can enhance the recruitment of patients with IBD into randomized controlled trials.

**Key Messages**

**What is already known?**

Recruitment for randomized controlled trials has traditionally required in-person visits with study staff, which can be time-consuming, costly, and limit the reach of study participants within a target population.

**What is new here?**

Bulk recruitment messaging through the electronic medical record (EMR) can reach more eligible participants faster and enroll them at higher rates than recruitment through in-person discussions with their inflammatory bowel disease (IBD) clinicians.

**How can this study help patient care?**

Tailoring the recruitment method to a study’s objectives, local clinical practices’ resources, and recruitment constraints can accelerate the development of therapies and expand patient participation, ultimately improving patient outcomes and IBD management.

## Introduction

Randomized controlled trials (RCTs) play a pivotal role in shaping patient care by providing evidence-based data for therapies that are used in clinical practice. RCTs are considered the gold standard for assessing the effectiveness of interventions. While randomization reduces the bias that may arise from differences in participant characteristics between study groups, recruiting an adequate number of participants within a specified timeframe to attain an appropriate sample size is a common challenge due to costs, time, and population sampling constraints.^1^

Optimizing recruitment strategies can be crucial for the overall success of clinical trials. Recruitment in inflammatory bowel disease (IBD) RCTs has historically been challenging, with enrollment rates < 1 patient/site/month.^2^ Recruitment into IBD trials includes an increasing number of trials competing for the same patient population pool,^2^ the substantial workload for physicians at participating recruitment sites,^2,3^ and increased time and cost constraints that limit clinic and staff research support capabilities.^2^

There are limited data directly comparing different recruitment strategies for RCTs in patients with IBD. Identifying the most effective approach for increasing IBD patient engagement in clinical trials can enhance research quality, create opportunities for advancements in the conduction of future pragmatic clinical trials, and improve patient care and outcomes. Thus, we aim to evaluate the effectiveness of traditional (in-person) versus decentralized (digital) recruitment methods in a multisite RCT for patients with IBD in the United States.

## Methods

In this secondary study, we analyzed the effectiveness of three recruitment strategies for the ADEPT trial (Addressing Disability Effectively with Psychosocial Telehealth; NCT05635292), an open-label, multicenter pragmatic RCT for adults with IBD. ADEPT was an effectiveness-implementation type 1 hybrid design trial^4^ conducted across five gastroenterology practices in the Crohn’s and Colitis Foundation’s Clinical Research Alliance in the United States: University of Pennsylvania, Vanderbilt University, University of Wisconsin, University of Arizona, and Gastroenterology Associates, Inc. Study candidates were invited to consent and complete a baseline eligibility survey online. Eligible participants with baseline moderate-to-severe IBD-related disability were invited to the ADEPT trial’s RCT for telehealth-delivered cognitive-behavioral therapy with a licensed psychologist versus usual care.

### Recruitment Strategies

Participants were recruited from February 2023 to October 2023 using one of three methods based on their clinical practice context and workflow (patient panel size, number of clinicians involved in recruitment, amount of time available to recruit patients, number of administrative staff available, etc), consistent with pragmatic clinical trial designs conducted in real-world contexts as per the Promoting Action on Research Implementation in Health Services (PARIHS) framework.^5^

#### (1) EMRs bulk messaging:

 Patients diagnosed with Crohn’s disease or ulcerative colitis, identified through International Classification of Diseases-10 (ICD-10) codes from IBD clinicians’ patient panels, received a study recruitment message sent in bulk via the EPIC electronic medical record (EMR) system at Sites A and B. At both sites A and B, greater than 90% of patients had active EMR messaging portals.

At Site B, only patients who had attended an office visit were identified as eligible for recruitment via EPIC messaging. Site E attempted to send one batch of EMR recruitment messages through eClinicalWorks (eCW), but since the messages were not ultimately delivered to patients the use of eCW in Site E was omitted from the analysis.

#### (2) Clinician discussion (clinician):

 Patients with IBD were approached by gastroenterologists who discussed the study with them in Sites C and E. These sites were also provided with 200 flyers that included information about the study’s objectives and the study website (plus a QR code) to distribute to patients.

#### (3) EMR bulk messaging and clinician discussion (hybrid)

combined the approaches of methods (1) and (2) and was used at Site D.

### Outcome Measures

The RE-AIM (Reach, Effectiveness, Adoption, Implementation, Maintenance) framework^6^ was utilized to evaluate each recruitment method’s recruitment rate, response rate, recruitment timeline, and the costs and maintenance of the recruitment methods. Quantitative data was captured on REDcap, and qualitative data was obtained through discussion with each site’s principal investigator(s).

### Statistical Analysis

Data was analyzed using descriptive statistics and Clopper–Pearson binomial confidence intervals for response rates with an a priori confidence level of 0.95.

## Results

A total of 3833 adults with IBD were approached across the 5 study sites for recruitment, and 630 participants were enrolled within 36 weeks (**Table 1**). The majority of patients identified as non-Hispanic White and approximately half were female, married, and had a Bachelor’s degree or higher education (**Supplementary Table**).

**Table 1.** ADEPT study sites and outcomes of their recruitment methods, including recruitment rate, response rate, recruitment timeline, direct costs, and description of time requirements

Sites	Description of site recruitment method	Recruitment rate (# enrolled / weeks)	Response rate (# enrolled / # approached)	Recruitment timeline	Direct costs	Description of time requirements
Overall	EMR, clinician, and hybrid methods	17.5 pt/wk 95% CI: [16.1–18.9 pt/wk]	16.4% (630/3833) 95% CI: [15.3%–17.7%]	36 wk (February–October 2023	$2800	N/A
Site A	EMR	17.6 pt/wk 95% CI: [15.0–20.2 pt/wk]	17.6% (176/1000) 95% CI: [15.3%–20.1%]	10 wk (July–September 2023)	$0	Automated EMR messaging system was already set up. Sending messages took <1 h. Bulk messages were sent in 2 cohorts across the entire recruitment timeline.
Site B	EMR	8.7 pt/wk 95% CI: [7.6–9.7 pt/wk]	13.0% (260/1985) 95% CI: [11.6%–14.7%]	30 wk (February–September 2023); bulk messages sent to groups of 100 to 300 patients (depending on size of IBD clinicians’ patient panels) each week.	$0	Automated EMR messaging system was already set up. Sending messages took 3 h. Bulk messages were sent across the entire recruitment timeline.
Site C	Clinician	5.1 pt/wk 95% CI: (4.1–6.3 pt/wk)	13.9% (82/592) 95% CI: (11.2%–16.9%)	16 wk (June–October 2023)	$100 for flyers	Flyers were preprinted. The study was discussed with approximately 37 IBD patients/wk for the full 16 wk of recruitment. Providers spent no more than 2 min discussing the study with each patient per visit, resulting in a total of approximately 20 h spent on recruitment.
Site D	Hybrid (EMR and clinician)	4.9 pt/wk 95% CI: (3.8–6.2 pt/wk) EMR: 1.1 pt/wk Clinician: 3.8pt/wk	Overall: 10.3% (64/616) 95% CI: (8.1%–13.1%) EMR: 7.0% (14/200) Clinician: 12.0% (50/416)	13 wk (July–October 2023)	$2500 for EMR; $100 for flyers	Automated EMR messaging system took 3 mo to set up administratively. EMR messages took 1 h to send. Bulk messages were sent across the entire 13 wk recruitment timeline. Flyers were preprinted. The study was discussed with approximately 32 IBD patients/wk for the full 13 wk of recruitment. Providers spent no more than 10 min discussing the study with each patient per visit, resulting in a total of approximately 69 h spent on recruitment.
Site E	Clinician	1.7 pt/wk 95% CI: (1.2–2.2 pt/wk)	2.6% (48/1820) 95% CI: (2.0%–3.5%)	28 wk (March–October 2023)	$100 for flyers	The study was discussed with approximately 65 IBD patients/wk for the full 28 wk of recruitment. Providers spent about 3 min discussing the study with each patient per visit, resulting in a total of approximately 91 h spent on recruitment.

The EMR method entails bulk patient messaging through the Epic messaging system. The Clinician method entails clinicians discussing the study with patients with the aid of flyers. The Hybrid method entails a combination of the EMR and Clinician methods. Only direct costs associated with implementing the recruitment method were calculated. All direct costs were attributed to printing flyers and setting up the EMR messaging system, if necessary. This analysis did not quantify the costs associated with personnel required for recruitment (eg, clinician cost of visit). Use of EMR bulk messaging resulted in the highest recruitment and response rates, along with the least costs and time required for recruitment, as compared to the Clinician and Hybrid methods.

ADEPT, Addressing Disability Effectively with Psychosocial Telehealth; EMR, electronic medical record; pt, participant

The study yielded an overall response rate of 16.4% [95% CI: 15.3%-17.7%] and an overall recruitment rate of 17.5 participants/week. Bulk EMR messaging alone (Sites A and B) had the highest recruitment rate (range: 8.7–17.6 participants/wk), highest response rate (range: 13.0–17.6%), lowest recruitment time (total 1–3 hours per site), and lowest direct costs ($0), as compared to the other recruitment methods (Clinician and Hybrid methods). Bulk EMR messaging resulted in significantly higher recruitment rates as compared to other recruitment methods. While there was no statistically significant difference in response rate by recruitment method, Site A did have a significantly higher response rate as compared to other sites. Site D incurred the highest start-up costs ($2000) due to information technology support to add bulk EMR messaging as a new recruitment modality. All sites using Clinician and Hybrid methods incurred a direct cost of $100/site for distributing study flyers. All sites plan to continue or explore using EMR messaging for future clinical trial recruitment.

## Discussion

When comparing recruitment rates, response rates, and time requirements associated with different methods for recruiting patients with IBD to a behavioral intervention RCT, EMR bulk messaging outperformed clinician discussions.

Recruitment costs varied depending on the existing local recruitment infrastructure. Although bulk EMR messaging approach streamlined recruitment by enabling efficient bulk messaging to a broad IBD population and reducing the burden on clinicians, our team encountered several challenges during implementation, many of which have been previously reported in studies on EMR-based recruitment.^7^ Setting up the necessary technical infrastructure required significant upfront costs, and training staff on how to use the EMR bulk messaging system effectively took additional time and effort. To our knowledge, we did not encounter issues with EMR data accuracy and patient preferences regarding research outreach messages, but prior studies have noted these as additional EMR-based challenges requiring manual review and adjustments.^7^

Although the ADEPT study had lower recruitment and response rates with in-clinic study discussions with clinicians, similar to another RCT on food-related quality of life,^8^ other RCTs with intensive interventions have had recruitment successes with clinician referrals (eg, exercise training regimens,^9^ multi-hour in-person education modules^10^). Of note, our staff felt that clinician-led recruitment placed a considerable strain on their workload, as it required significant time and effort. This challenge could hinder the scalability of this recruitment approach.

Limitations of this study include the secondary analysis of the available and best-suited recruitment method(s) used by each clinical practice to recruit among IBD clinicians’ IBD patient panels, potentially resulting in selection bias and limited generalizability. Nonetheless, this reflects the purpose and design of a pragmatic clinical trial in real-world settings where patient care is delivered, including academic university specialty clinics and community and private practices. Also, feedback from patients regarding their perception of recruitment methods was not included in this study, as the focus was on optimizing recruitment and response rates. However, future studies could benefit from incorporating this qualitative dimension to identify patient-preferred recruitment approaches.

## Conclusion

Effective and efficient participant recruitment can impact time, costs, and the overall success of RCTs. Protocolizing, analyzing, and reporting recruitment strategies and the design and scope of future RCTs can increase patient engagement in clinical trials and improve research quality. In the ADEPT trial, EMR bulk messaging reached more eligible patients faster than in-person and hybrid recruitment methods. This strategy can be particularly valuable at sites where patients are accustomed to using the EMR portal to send and receive communications about their healthcare and hence are more likely to see, read, and respond to EMR bulk messaging.

## Supplementary Material

otaf033_suppl_Supplementary_Materials

## Data Availability

Data not publicly available, but can be made available upon reasonable request to the corresponding author.
