# Supplementary material for: Patient Recruitment Strategies for Behavioral Clinical Trials in Adults with Inflammatory Bowel Diseases: An Analysis of the ADEPT (Addressing Disability Effectively with Psychosocial Telehealth) Randomized Controlled Trial
Source: Crohns Colitis 360. 2025 May 2;7(2):otaf033. doi: 10.1093/crocol/otaf033 (PMC12082830; doi:10.1093/crocol/otaf033)
Supplement: otaf033_suppl_Supplementary_Materials [file otaf033_suppl_supplementary_materials.docx]

**Supplementary Table: Patient Demographics (n=630)**

| **Variable** | **Total**  **n=630 (100%)** |
| --- | --- |
| **Age (in years), mean (SD)** | 43.0 (15.2) |
| **Race, n (%)^a^**  White  Black  Native  Asian  Hawaiian and/or Pacific Islander  Unknown  Prefer not to answer  Missing data | 486 (77.1%)  48 (7.6%)  3 (0.5%)  14 (2.2%)  1 (0.2%)  3 (0.5%)  14 (2.2%)  79 (12.5%) |
| **Ethnicity, n (%)**  Hispanic or Latino  Not Hispanic or Latino  Prefer not to answer  Missing data | 24 (3.8%)  508 (80.6%)  19 (3.0%)  79 (12.5%) |
| **Gender, n (%)**  Male  Female  Prefer not to answer  Missing data | 203 (32.2%)  345 (54.8%)  3 (0.5%)  79 (12.5%) |
| **Marital Status, n (%)**  Married or lives with partner  Not married or not living with anyone  Divorced  Widow  Missing data | 339 (53.8%)  156 (24.8%)  36 (5.7%)  10 (1.6%)  89 (14.1%) |
| **Education Status, n (%)**  Elementary School  Highschool diploma  Sub-bachelor or vocational diploma/certificate  Associate’s degree  Bachelor’s degree  First professional degree  Post-bachelor’s diploma/certificate  Master’s degree  Doctorate or advanced professional degree  Different professional course completed  No school or education completed  Missing data | 3 (0.5%)  102 (16.2%)  42 (6.7%)  50 (7.9%)  156 (24.8%)  3 (0.5%)  8 (1.3%)  125 (19.8%)  56 (8.9%)  4 (0.6%)  2 (0.3%)  79 (12.5%) |

*^a^Patients were able to select more than one race. For this reason, percentage totals exceed 100%.*
